# Changes in intestinal microflora and its metabolites underlie the cognitive impairment in preterm rats

**DOI:** 10.3389/fcimb.2022.945851

**Published:** 2022-08-19

**Authors:** Xiang Lu, Zhengyang Xue, Yu Qian, Shanjie Wei, Yu Qiao, Wen Zhang, Hongyan Lu

**Affiliations:** ^1^ Pediatrics, Affiliated Hospital of Jiangsu University, Zhenjiang, China; ^2^ School of Medicine, Jiangsu University, Zhenjiang, China

**Keywords:** preterm delivery, cognitive impairment, intestinal microflora, short-chain fatty acids, microbiota-gut-brain axis, abundance

## Abstract

**Background:**

The brain development of preterm infants is easily affected by various adverse extrauterine factors and complications, resulting in abnormal neurological and cognitive development. Recent studies have found that there is a significant correlation between intestinal microbial changes and cognitive behavior. Nevertheless, the correlation between the cognitive impairment and abnormal changes of intestinal microflora in the preterm newborn has been rarely elucidated.

**Aim:**

To analyze the differences of fecal intestinal flora, short chain fatty acids (SCFAs) and microbiota-gut-brain axis (MGBA)-related serum factors between preterm birth with and without cognitive impairment.

**Methods:**

Healthy female rats (body weight 410 ± 40 g) of 16-17 days of gestation were selected for the establishment of preterm cognitive impairment model and screened by Morris water maze navigation experiments. The pathological change of rat hippocampus was confirmed by HE staining. The abundance of fecal intestinal microflora was determined by 16sRNA sequencing, while the contents of fecal SCFAs were examined by gas chromatography.

**Results:**

Compared with the control group, the cognitive impairment group had decreased abundance and diversity of intestinal microflora and increased abundance of *Proteobacteria* at the level of phylum. While the abundances of *Alistipes*, *Bacteroides*, *Prevotella*, and *Lactobacillus* decreased significantly at the level of order, family, and genus, the abundances of *Staphylococcaceae*, *Enterococci*, *Psychrobacter*, and *Oligella* increased significantly. Moreover, the levels of total SCFAs and acetic acid in the disease group were significantly lower. The fecal abundance of acetic acid was positively correlated with that of *Lactobacillaceae* or *Peptostreptococcaceae*, and negatively correlated with that of *Aerococcaceae*, and *Alcaligenaceae* in disease rats. Furthermore, cognitive impairment caused significantly decreased levels of 5-HT, GABA, and BDNF, and increased levels of GR, CRH, IL-6, and TNF-α in rat blood.

**Conclusion:**

Alterations in intestinal microflora structure and the abundances of SCFAs contributed substantially to the cognitive impairment in preterm rats, which was associated with significant changes in MGBA-related soluble factors.

## Introduction

Preterm infants leave their mothers’ bodies prematurely, and hence, the development of their brains is susceptible to damages caused by various adverse extrauterine factors and complications, which can result in abnormal cognitive development (Spittle et al., 2015; Einspieler et al., 2016). Cognitive impairment has been reported in about 40% of preterm infants with very low body weight, and it exerts a negative impact on school education and daily life of the preterm children (Sobaih, 2018). Recent studies have demonstrated a significant correlation between the change in the intestinal microflora and cognitive development (Gareau, 2016; Bruce-Keller et al., 2017; de Weerth, 2017). Intestinal microflora consists of a large number of species and is a general term for a variety of microbial communities coexisting with the host in the intestinal tract (Lynch and Pedersen, 2016). It was found that the germ-free mice exhibited anxiolytic behavior in comparison to the specific pathogen-free (SPF) mice (Neufeld et al., 2011). When the germ-free mice were implanted with normal intestinal microflora, they reduced anxiolytic behavior and were similar active as the SPF mice (Neufeld et al., 2011), suggesting that the intestinal microbial colonization-stimulated nerve cells were involved in behavioral activity and anxiety.

Recently, a microbiota-gut-brain axis (MGBA) has been proposed to explain the biological and physiological basis of psychiatric, neurodevelopmental, age-related, and neurodegenerative disorders (Cryan et al., 2019). The microbiota and the brain communicate with each other *via* various routes in the enteric nervous system, the hypothalamic-pituitary-adrenal (HPA) axis, and the immune system (Liang et al., 2018; Cryan et al., 2019). Several soluble factors, including the neurotransmitters 5-HT (5-hydroxytryptamine) and GABA (gamma-aminobutyric acid), GR (Glucocorticoid) and CRH (Glucocorticoid-releasing hormone) released from the HPA axis, the neurotransmitter modulator BDNF (brain-derived neurotrophic factor), as well as the pro-inflammatory cytokines IL-6 (interleukin-6) and TNF-α (tumor necrosis factor-α), were reported to be involved in regulating the MGBA (Carabotti et al., 2015; Zhao et al., 2018; Sinagra et al., 2020; Sun et al., 2020). However, whether and how the MGBA in preterm infants with impaired cognitive function is dysregulated remains to be extensively explored.

The intestinal microflora is found to regulate the development and function of microglia and astrocytes through its metabolites, termed as short-chain fatty acids (SCFAs) (Carabotti et al., 2015; Gareau, 2016; Lynch and Pedersen, 2016). SCFAs, which are mainly composed of acetic acid, propionic acid, and butyric acid, are formed by intestinal bacteria through carbohydrate fermentation in the colon and the distal part of the small intestine (Carabotti et al., 2015; Gareau, 2016; Lynch and Pedersen, 2016). Erny *et al.* showed that SCFAs promote the development and regulate the activity of microglia, affect the differentiation of oligodendrocyte precursor cells and the formation of the myelin sheath, and enhance the immune defense function of the brain (Erny et al., 2015). A study on the adult inflammatory bowel disease demonstrated that early life events could change the development of microbial population after birth, leading to the imbalance of gut-brain communication, thus altering the brain development and behavior (Collins et al., 2012). Moreover, in the early postnatal period, the decrease in the abundance and diversity of intestinal probiotics and a decrease in the content of SCFAs in metabolites caused anxiety and cognitive behavioral changes in mice (Sudo et al., 2004). These abnormal changes in the intestinal flora and SCFA metabolites are detected in psycho-neurodegenerative diseases such as depression and autism (Zhu et al., 2020). Furthermore, probiotics improve the host anxiety, depression, cognitive function, autism, stress response, and other neurological disorders (Zhu et al., 2020), suggesting that intestinal flora and SCFA metabolites are crucial for brain development and cognitive function formation. Nevertheless, the correlation between the cognitive impairment and abnormal changes of intestinal microflora in the preterm newborn has been rarely elucidated.

In this study, we established an animal model of preterm rats with cognitive impairment and compared the structural characteristics of fecal intestinal microflora from the disease rats and control rats. In addition, the correlation between the abundance of fecal SCFAs and intestinal representative bacteria, as well as the changes of MGBA-related soluble factors in serum, were explored to further understand the mechanisms underlying the cognitive impairment in preterm rats from the perspective of gut-brain axis.

## Materials and methods

### Establishment of cognitive impairment preterm rat model

Animal disease models are the basis for studying disease pathogenesis and prevention. Therefore, establishing a reasonable model of cognitive impairment in premature rats is of great significance for the study of pathogenesis. Peripheral inflammation induced by intraperitoneal injection of lipopolysaccharide (LPS) can act on the brain parenchyma and activate microglia in the central nervous system (CNS) to stimulate the synthesis of inducible nitric oxide synthase (iNOS) (Teeling and Perry, 2009; Kettenmann et al., 2011), thereby synthesizing a large amount of nitric oxide (NO), eventually leading to neuronal death and cognitive impairment (Yu et al., 2005). In the research, healthy pregnant Sprague Dawley (SD) female rats with specific pathogen-free (SPF) grade, 16-17 days in gestation (The due date is 22 days), body weight 410 ± 40 g, were purchased from Experimental Animal Center of Jiangsu University (Zhenjiang, China) under a License Number SYXK (Su) 2013-0036. The model of preterm rats with cognitive impairment was established as described previously (Muccigrosso et al., 2016). In the experimental group, the pregnant SD rats were given intraperitoneal injection of LPS (Sigma-Aldrich, St. Louis, MO, USA; 330µg/kg body weight each time per day) for 2 consecutive days between 16 and 17 days after pregnancy. In the control group, the pregnant SD rats received equal amounts of phosphate buffered saline (PBS) instead of LPS. Cesarean section was performed on day 20 of gestation (22 days at full term the mother rats could not survive after caesarean section), and the premature rats were randomly fed by surrogate mothers for 30 days. The number of pups per litter varies. There are 15 pregnant rats in our experimental group injected with LPS, and 2 premature rats in each litter were selected to participate in the follow-up experiments; the control group had 8 pregnant rats, and 1-2 premature rats were selected from each litter to participate in the follow-up experiments. After that, we obtained 30 premature rats in the model group and 15 premature rats in the control group. Totally 45 rats were used in this study and 9 surrogate mothers were adopted. Each surrogate mother fed 5 premature rats, and 30 rats in the model group and 15 rats in the control group were obtained and numbered. Rats were given food and clean drinking water. The feeding environment with a room temperature of (25.0 ± 0.5) °C and a normal light/dark cycle of 12 h/12 h was maintained. At 30 days after birth, rats in both groups were evaluated by positioning navigation experiment in Morris water maze. All animal experiments involving the use of rats in this study were approved by the Institutional Animal Care and Use Committee of Jiangsu University, and all experiments were performed in accordance with relevant guidelines and regulations.

### Morris water maze (MWM) assay

The spatial learning and memory function of preterm rats was evaluated by the positioning navigation experiments in the MWM assay. On the day before the experiment, 45 preterm rats (30 days old) were placed in the pool with a diameter of 1.6 m and a water depth of 1.2 m. At the age of 1 month, SD rats are about the same as human 2 ~ 5 years old, that is early childhood. At this time, whether the development of cognitive function is normal or not can be fully demonstrated. The animals were allowed to swim freely for 2 min to familiarize them with the maze environment. After the start of the experiment, the hidden platform was placed 2 cm below the water surface, and each rat was trained 4 times on reaching the hidden platform from the fixed points of different quadrants every day at an interval of 15 s. The training time was 10:00–16:00 daily. The environment and water temperature were maintained at 20°C. The eye-catching signs were placed on the four walls of the water maze at fixed positions. The experimenter wore the same clothes every day. The rats from each group were placed into the pool from the four fixed starting points of the pool wall sequentially. The video recording system automatically recorded the time when the rats found the platform and the swimming path. If the rats could not find the platform within 2 min, the experimenter led them to the platform and rested for 10 s. The incubation period was recorded as 2 min. The average incubation period of 4 training sessions/day was considered as the score of the day. After four times of training, the rats were dried with a towel and placed back into the ventilated cage. The same training was conducted for 5 consecutive days. In this experiment, the cognitive function of rats was evaluated by positioning navigation test. The longer the time to find the platform, the lower the cognitive function.

### Hematoxylin-eosin (HE) staining of hippocampal tissues

After collection of blood samples, rats were euthanized with CO_2_ inhalation. The whole brain was removed after perfusion with PBS and subsequent 4% paraformaldehyde. Brain samples were fixed, dehydrated, paraffin-embedded, sectioned, and stained with HE staining with the standard method. The morphological changes in the hippocampal cells were observed and evaluated by experienced pathologists.

### Group assignment of preterm rats

**Figure 1:**
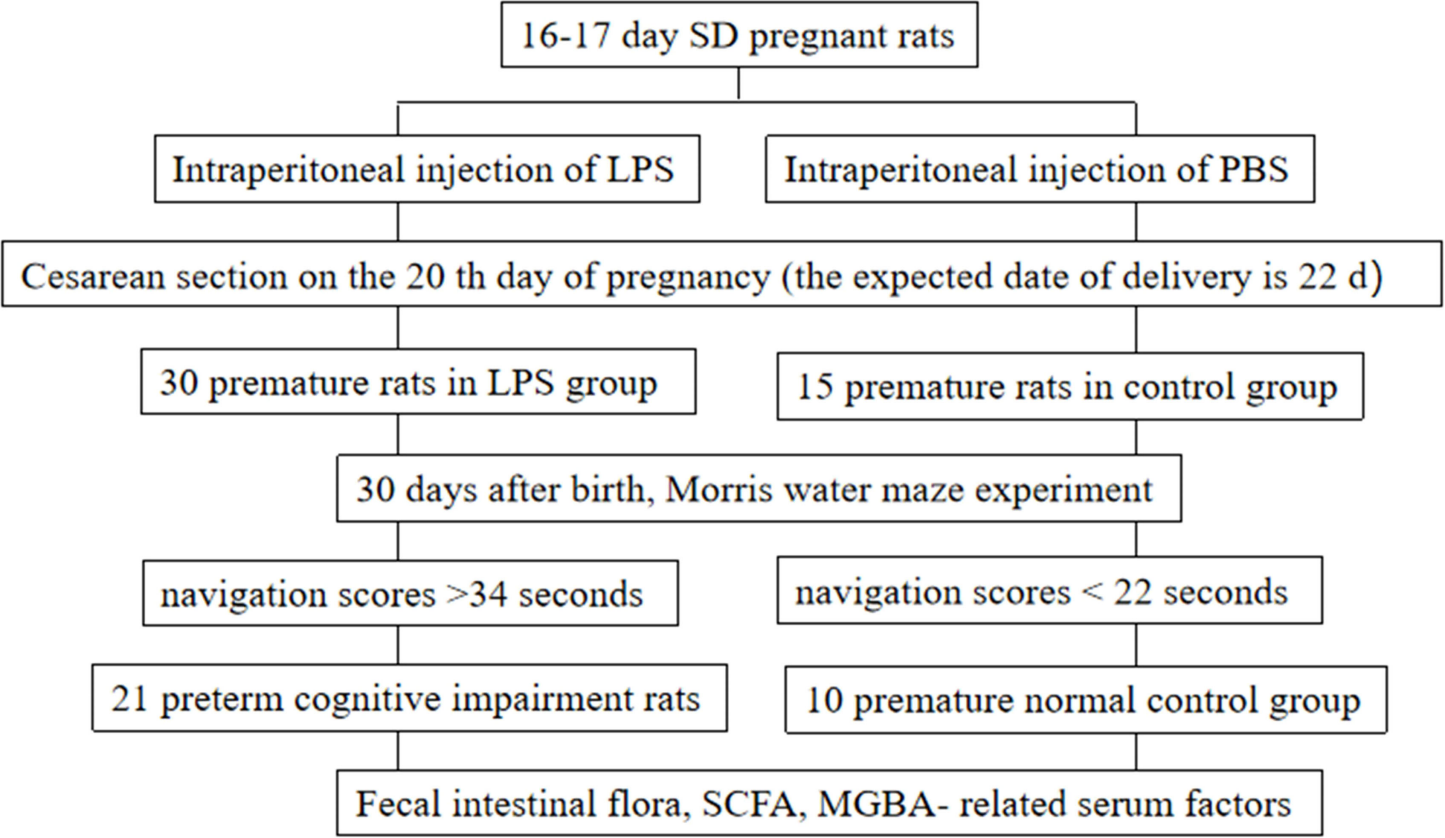
Experimental flow chart of the rat groups assignment to describe the experimental design.

In the normal control group, the structure of hippocampal neurons was clear, the size was normal, and the nuclei were stained with light blue; In preterm cognitive impairment group, the number of hippocampal neurons decreased significantly, the cells were arranged irregularly, and a large number of neurons were degenerative and necrotic. According to the results of HE staining in the hippocampus and the navigation tests, out of the 30 model rats, 21 rats with abnormal morphological changes in the hippocampal cells and with lowest navigation scores longer than 34 seconds were assigned to the preterm cognitive impairment group. In addition, out of the initial 15 rats of the control group, 10 rats with normal morphology of hippocampal cells and the highest navigation scores less than 22 seconds were assigned to the normal control group. After renumbering, the intestinal flora of these 31 grouped rats (21 rats in the experimental group and 10 rats in the control group) was examined. We designed a flow chart of the rat groups assignment to describe the experimental design (**Figure 1**).

### 16S rRNA sequencing of fecal samples

After sterilization of rat anus with 75% alcohol cotton ball, feces samples were collected from the preterm rats at 30 days after birth in each group. The fecal samples were placed into sterile 5 mL centrifuge tubes, and the tubes were sealed and stored at - 80°C. The QIAamp DNA Stool Mini Kit (QIAGEN, Hilden, Germany) was used to extract genomic DNA from the samples. The DNA concentrations and purity were evaluated with the Onedrop Instrument (Nanjing Wuyi Technology Co., Ltd., Nanjing, China) and agarose gel electrophoresis. An equivalent of 2.5 ng diluted genomic DNA was used as a template for PCR amplification together with the 16S V4 universal primer (515F/806R) with barcode (Life Technologies, Carlsbad, CA, USA) and GoTaq^®^ Hot Start Colorless Master Mix high fidelity enzyme (Promega). MD Fluorescence Quantitative Detector (Molecular Device, San Jose, CA, USA) was used for the quantitative detection of DNA concentration in PCR products, and the amplified products were purified using Qiaquick^®^ PCR purification kit (QIAGEN).

The second round of amplification was carried out using Illumina bridge PCR compatible primers (Illumina, San Diego, CA, USA) on the recovered products that were subsequently detected by PicoGreen fluorescence quantification (Promega) and Agilent 2200 TapeStation electrophoresis platform (Santa Clara, CA, USA). Illumina Miseq sequencer (Illumina) was used for onboard sequencing. The original data from high-throughput sequencing were separated according to sample barcode using vendor software from Illumina. Primer sequences of paired-end reads were trimmed using Trim Galore v0.6.5 (https://www.bioinformatics.babraham.ac.uk/projects/trim_galore) with options ‘–phred33 –length 35 –stringency 3 –fastqc’. Quality control reports were then integrated using MultiQC (Ewels et al., 2016) with default parameters. Ribosomal database project (RDP, Release 11.5) classifier 2.3 (https://rdp.cme.msu.edu/) was applied, and the Silva database v138 (https://www.arb-silva.de) was used for sequence alignment to determine the classification level (boundary, phylum, class, order, family, genus, species) of each sequence. Mothur (https://mothur.org/) was employed to divide the operational taxonomic units (OTUs), and taxonomic operating units with 97% sequence similarity were considered as the standards. Finally, the OTU abundance spectrum was generated according to the number of sequences.

### Determination of fecal SCFAs by gas chromatography

A 25% w/v of metaphosphoric acid solution was mixed with 0.6464 g crotonic acid in a 100-mL volume. Then, 0.9100 g acetic acid, 0.3700 g propionic acid, 0.1765 g butyric acid, 0.1765 g isobutyric acid, 0.1985 g valeric acid, and isovaleric acid, were separately solubilized in 100 mL double distilled water. An equivalent of 1 g fecal sample was diluted in 5–10 volumes of distilled water. A volume of 1 mL of the supernatant was mixed with 0.2 mL crotonic acid metaphosphate mixture and stored at -20°C overnight, followed by centrifugation at 12000 rpm for 10 min. A volume of 0.2–1.0 μL filtrate was directly injected into the chromatography column using a 1.0 μL microinjector. Similarly, 0.2 mL of metaphosphoric acid and the crotonic acid mixture were added into a 1-mL standard sample, and the retention time of acetic acid, propionic acid, butyric acid, isobutyric acid, valeric acid, and isovaleric acid on the column was determined with the GC-14B Gas chromatograph instrument (Shimadzu, Kyoto, Japan). The relative correction factors of organic acids, such as acetic acid, propionic acid, and butyric acid, were calculated by the respective concentration and peak area of the standard sample and internal standard crotonic acid. Then, the concentrations of acetic acid, propionic acid, and butyric acid in each sample were estimated according to the weight (or concentration) of each acid, which was directly proportional to the peak area. The following formula was used: acid concentration (mM) = (area value of a certain acid peak in the sample × area of a certain acid standard sample × concentration of a standard acid sample)/(area value of crotonic acid in the sample × area of an acid peak in the standard sample).

### Rat serum collection and quantification of soluble factors

After the MWM experiments, rats were anesthetized with ketamine (80 mg/kg body weight) and dexedetomidine (0.5 mg/kg body weight) through intraperitoneal injection. Blood samples were collected from each group of rats through cardiac puncture. Serum was obtained by centrifugation of blood samples at 10, 000 g at 4°C for 10 min, and stored at -20°C. The levels of BDNF, CRH, GR, 5-HT, GABA, IL-6, and TNF-α in rat serum were determined by enzyme-linked immunosorbent assay (ELISA) kits (Elabscience Biotechnology Co.,Ltd, Wuhan, China) following the manufacturer’s protocols.

### Statistical analysis

SPSS 19.0 statistical software was used for data analysis. The measurement data were expressed as mean ± standard deviation ( $\bar{x}$ ± s). The comparison between the two groups of samples was conducted by two samples *t*-test and median (interquartile spacing) [M (P25, P75)]. The abundance of species and SCFAs of intestinal flora between the two groups were compared by Mann–Whitney U test. Principal component analysis (PCA) was used to decompose the variance between the two groups, and the main elements and structures of the data were extracted after dimensionality reduction. Spearman’s rank correlation was used in the correlation analysis. A *P<* 0.05 indicated statistical significance.

## Results

### Preterm rats in the normal control group and cognitive impairment group displayed significantly differed cognitive ability in the MWM test and pathological changes of hippocampal neurons

To establish the model of preterm rats with cognitive impairment, we treated the female rats at a gestation stage of 16-17 days with LPS to induce damages to fetuses and obtained the preterm rats by delivery with cesarean section. The rats in the control group were from the preterm rats delivered from the mother rats mock-treated with PBS. At day 30 after birth, rats delivered from the LPS-treated mothers had an average body weight of 109.29 ± 8.15 g, while rats delivered from the PBS-treated mothers had a comparable average body weight (106.10 ± 6.61 g). Although these two groups of rats did not demonstrate significant difference (t = 1.08, *P* > 0.05) in body weight, they did show difference in cognitive ability in the position navigation experiments of the MWM test. The average time needed to find the platform for rats in the cognitive impairment group was 40.27 ± 4.61 s, which was significantly longer (t = 13.29, *P*< 0.05) than that for rats in the normal control group (17.75 ± 3.94 s) (**Supplementary Table 1**)(**Supplementary Material**).

**Figure 2:**
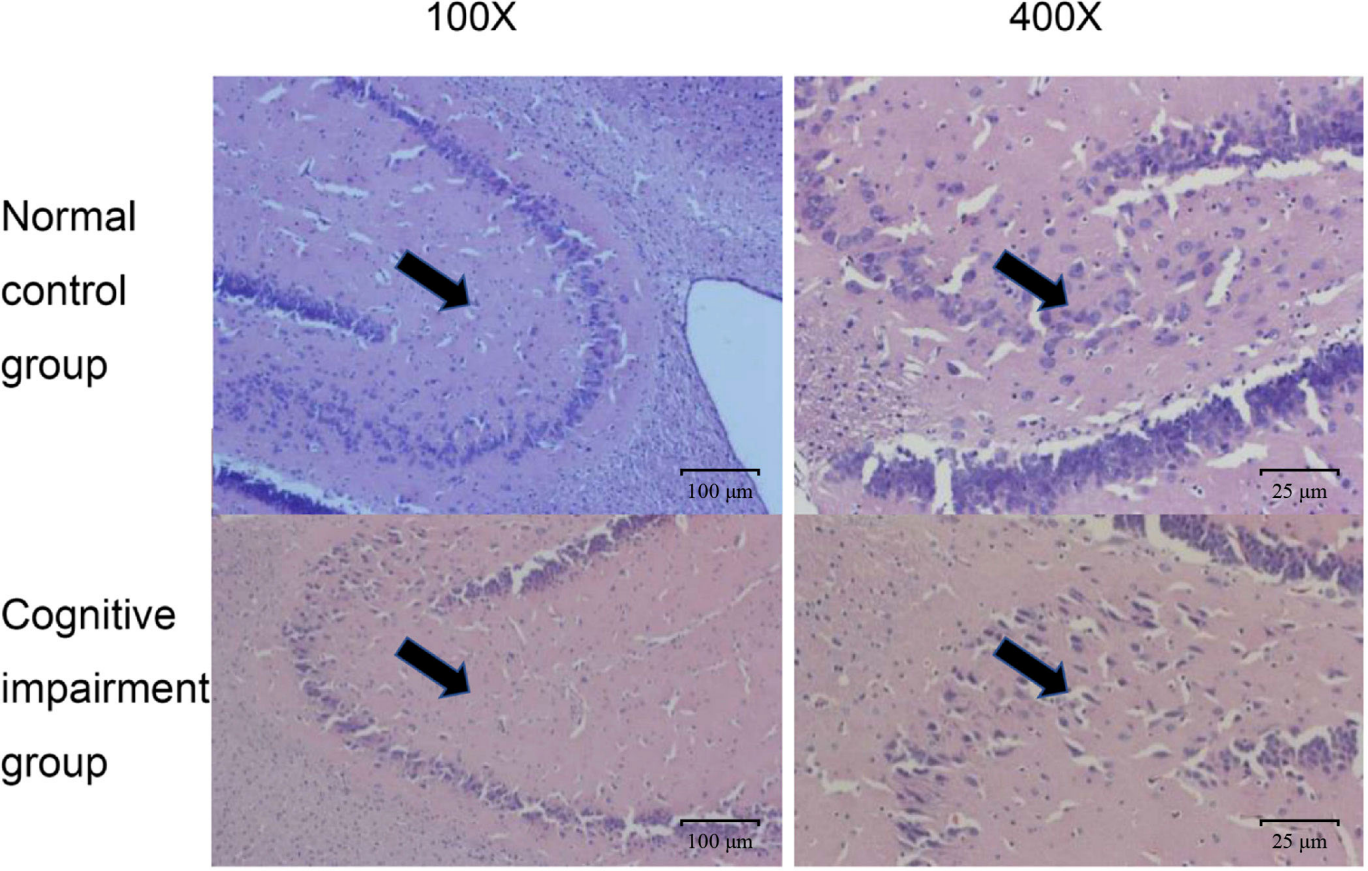
Comparison on pathological changes of hippocampal neurons from rats in the normal control group and the cognitive impairment group. Representative images of HE staining results from the normal control group and the cognitive impairment group. The hippocampal neurons were clear in structure, normal in size, and stained light blue; while those in the cognitive impairment group were not arranged orderly, largely degenerated, and necrotic. Magnification, 100 × and 400 ×, as indicated.

In addition, we also confirmed the morphological and structural differences of hippocampal neurons in rats of these two groups, since hippocampus is the key area of the brain responsible for emotional and cognitive functions (Lisman et al., 2017). As shown in **Figure 2**, hippocampal neurons in the normal control group had normal sizes, and the nuclei were round, oval, and stained light blue. Whereas, in the preterm cognitive impairment group, hippocampal neurons were not arranged orderly, and a large number of neurons were degenerated and necrotic. Moreover, the cognitive impairment group appeared to have a decreased number of hippocampal neurons in comparison to the normal control group.

### Differences of intestinal microflora diversity between the two groups

**Figure 3:**
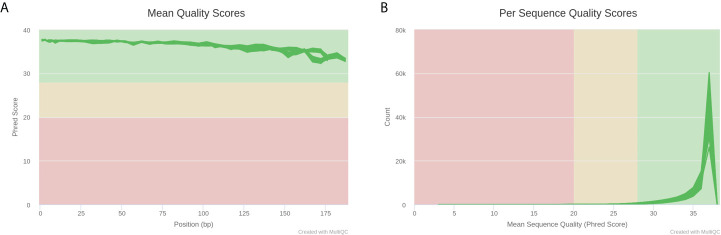
MultiQC report plots of 31 libraries after Trim Galore quality control. **(A)** Average sequencing quality of bases at each position in each read. **(B)** Number of reads with average quality score.

**Figure 4:**
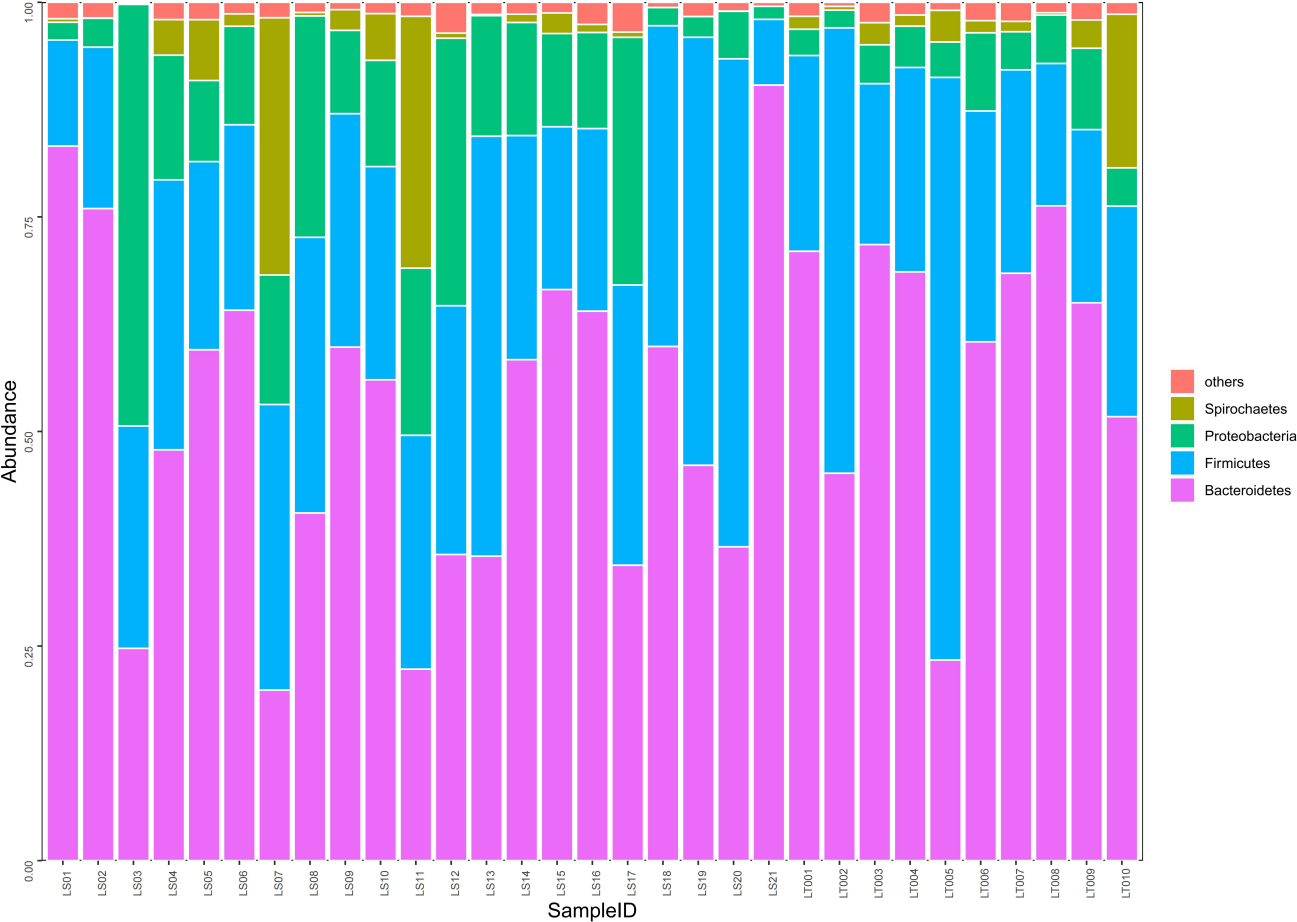
Comparison on the distributions of intestinal microflora at the phylum level in two groups of rats. Fecal samples from the preterm rats at day 30 after birth were subjected to 16S rRNA sequencing. The relative abundances of intestinal microflora including *Bacteroidetes*, *Firmicutes*, *Proteobacteria*, and *Spirochaetes* were summarized in the bar graph. The abscissa represents all the individual samples, and the ordinate represents the relative abundance of each phylum. LT001–LT010, the normal control group; LS1–LS21, the cognitive impairment group.

**Figure 5:**
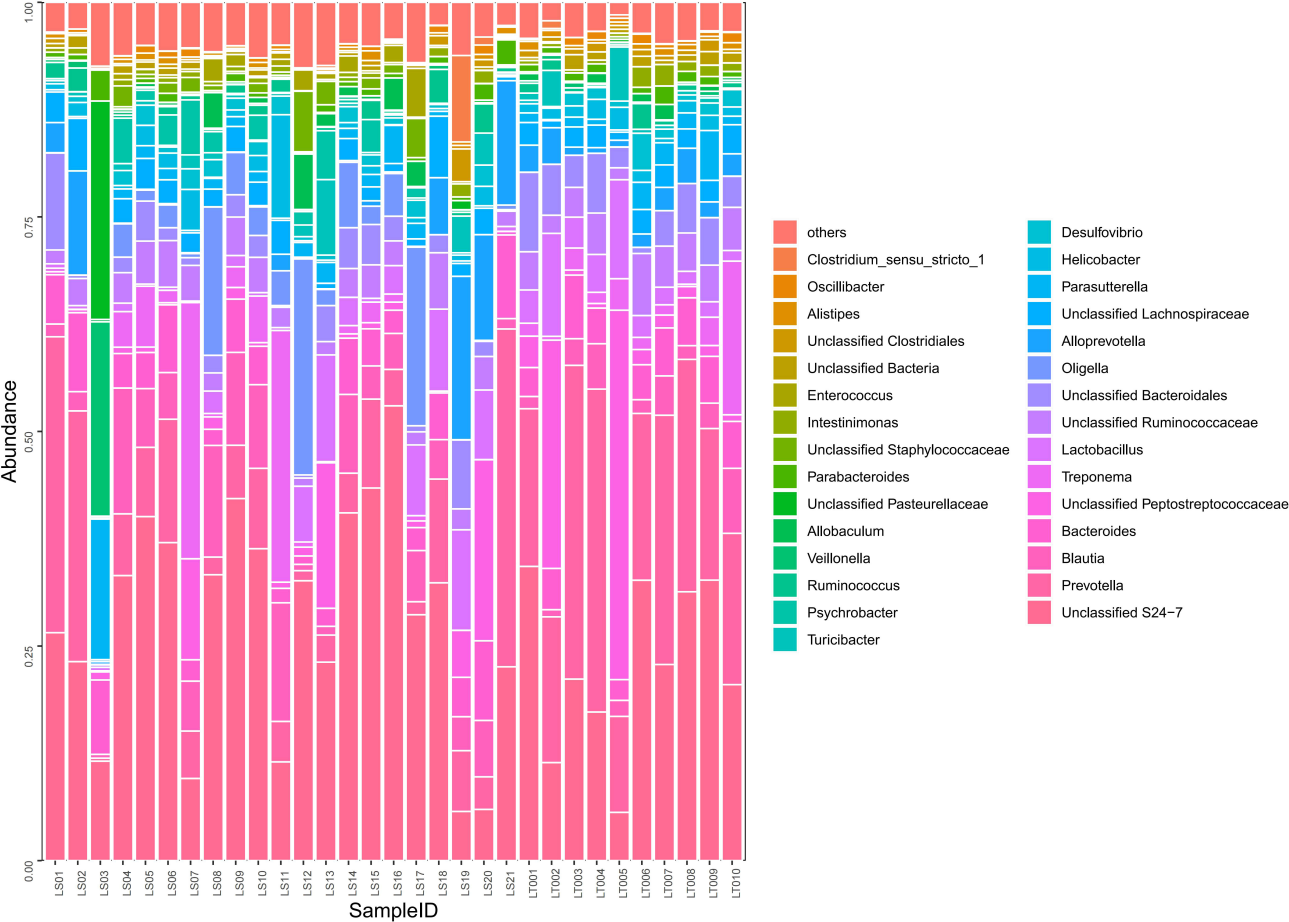
Comparison on the distributions of intestinal microflora at the order, family, and genus levels in two groups of rats. Fecal samples from the preterm rats at day 30 after birth were subjected to 16S rRNA sequencing. The relative abundances of the indicated intestinal bacterial orders, families, and genera were summarized in the bar graph. The abscissa represents all the individual samples, and the ordinate represents the relative abundance of each genus. LT001–LT010, the normal control group; LS1–LS21, the cognitive impairment group.

Next, we selected fecal samples from 21 rats of the cognitive impairment group with abnormal hippocampal structure and the lowest scores in MWM navigation test, as well as 10 rats of the normal control group with normal hippocampal structure and the highest scores in MWM navigation test and performed 16S rRNA sequencing. These 31 libraries generated a total of 4,628,290 raw reads on the Illumina MiSeq platform. After quality control and trimming of adapter sequences using Trim Galore software, 4,621,736 clean reads were subsequently obtained (**Supplementary Material**). The quality report acquired using MultiQC is shown in **Figure 3**. The comparisons of intestinal microflora in terms of diversity and abundance suggested that rats with cognitive impairment had significantly altered profiles of intestinal microflora (**Figures 4**, **5** and **Supplementary Tables 2**–**4**).

The richness and diversity of flora in a specific region or ecosystem are often measured using the Chao1 index, ACE index, Shannon index, and Simpson index (Morris et al., 2014; Chen et al., 2020). The Chao1 and ACE indexes are mainly used to estimate species richness. The larger the Chao1 and ACE indexes are, the richer the flora in the representative sample is. The Shannon and Simpson indexes are mainly used to evaluate species diversity. The larger the Shannon index is, or the smaller the Simpson index is, the higher the flora diversity in the representative sample is (Morris et al., 2014; Chen et al., 2020). Compared to the normal control group, the cognitive impairment group had significantly (*P*< 0.05) lowered indexes of bacterial richness, including both the Chao1 index and the ACE index. In addition, the Shannon index, which represents the bacterial diversity, was also significantly lower (P< 0.05) in the cognitive impairment group. However, the Simpson index did not differ significantly between the two groups (*P* > 0.05) (**Supplementary Table 2**). Taken together, intestinal flora from the preterm rats with cognitive impairment had significantly reduced richness and diversity, as evidenced by marked alterations in three out of four indexes.

### Differences in intestinal microflora at the phylum level between the two groups

Our sequencing results demonstrated that about 98.4% of the intestinal bacteria in the samples consisted of *Bacteroidetes*, *Firmicutes*, *Proteobacteria*, and *Spirochaetes*. Among these phyla, *Bacteroidetes* and *Firmicutes* are the dominant groups of intestinal flora in rats (**Figure 4**). Interestingly, we found that no significant difference in the abundance of *Bacteroidetes*, *Firmicutes*, and *Spirochaetes* between the cognitive impairment group and the normal control group was identified (*P* > 0.05), while the abundance of *Proteobacteria* increased significantly in the cognitive impairment group (*P*< 0.05) (**Figure 4** and **Supplementary Table 3**).

### Difference in intestinal microflora at the levels of order, family, and genus between the two groups

A total of 97 species of bacteria at the level of order, family, and genus were identified through sequencing. Compared to the normal control group, the cognitive impairment group displayed significantly higher abundance in harmful bacteria including *Oligella*, *Psychrobacter*, *Staphylococcaceae*, and *Enterococci* (*P*< 0.05), while the abundance of probiotics such as *Prevotella*, *Lactobacillus*, *Bacteroides*, and *Alistipes* were significantly lower in the cognitive impairment group (*P*< 0.05) (**Supplementary Table 4** and **Figure 5**).

**Figure 6:**
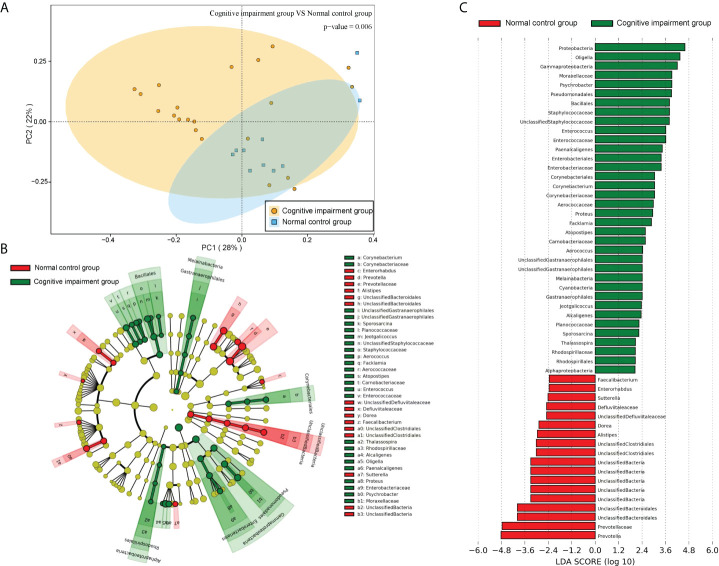
The microbiota structures of fecal samples from the normal control group and the cognitive impairment group were compared. **(A)** The PCA result revealed a significant difference in gut bacterial structure between the two groups. The two principal component scores accounted for 28 (PC1) and 22% (PC2) of total variations, respectively. Each symbol represents an individual sample. **(B, C)** The dominant floras of the two groups were analyzed by LEfSe (Linear discriminant analysis Effect Size). **(B)**Taxonomic representation of statistically and biologically consistent differences between fecal samples from the normal control group and the cognitive impairment group. Differences are represented in the color of the most abundant class (red, the normal control group; green, the cognitive impairment group; yellow, non-significant). Each circle’s diameter is proportional to the taxon’s abundance. **(C)** Histogram of the LDA scores computed for features differentially abundant between the two groups. The LDA threshold is 2.0.

In addition, further PCA analysis showed that the distance between the sample points of the normal control group was shorter (**Figure 6A**), suggesting that the intestinal flora of the normal control group was similar. However, the distance between the sample points of the cognitive impairment group was larger (**Figure 6A**), indicating that the intestinal flora of the preterm rats differed markedly upon LPS-induced cognitive brain injury. Moreover, the distance between the two groups was also large, implying that the composition of the intestinal flora of the two groups was significantly different (PERMANOVA, *P* =0.006). Furthermore, we analyzed the difference in the abundance of the microbiota components using LEfSe and screened the dominant floras. It was found that the dominant floras in the feces of the cognitive impairment group were *Proteobacteria*, *Oligella*, *Gammaproteobacteria*, and *Moraxellaceae*; while the dominant fecal floras in the control group were *Prevotella*, *Prevotellaceae* and *Bacteroidales* (**Figures 6B, C**).

### Preterm rats in the normal control group and cognitive impairment group displayed different profiles of short-chain fatty acids in feces

We further investigated whether the change in abundance of fecal SCFAs was associated with differed intestinal microflora profiles and cognitive impairment in preterm rats through gas chromatography analyses of fecal samples. The results showed that fecal SCFAs were mainly composed of formic acid, acetic acid, propionic acid, butyric acid, and valeric acid, while over 95% of SCFAs were acetic acid, propionic acid, and butyric acid. According to results with the Mann–Whitney U test, the level of total SCFAs and acetic acid in the cognitive impairment group was significantly lower than that in the normal control group (*P<* 0.05), while no significant difference in levels of other SCFAs (including propionic acid, isobutyric acid, butyrate, isovaleric acid, and valeric acid) between the two groups was identified (**Supplementary Table 5**).

### Correlation analysis between the abundance of fecal acetic acid and representative bacteria

**Figure 7:**
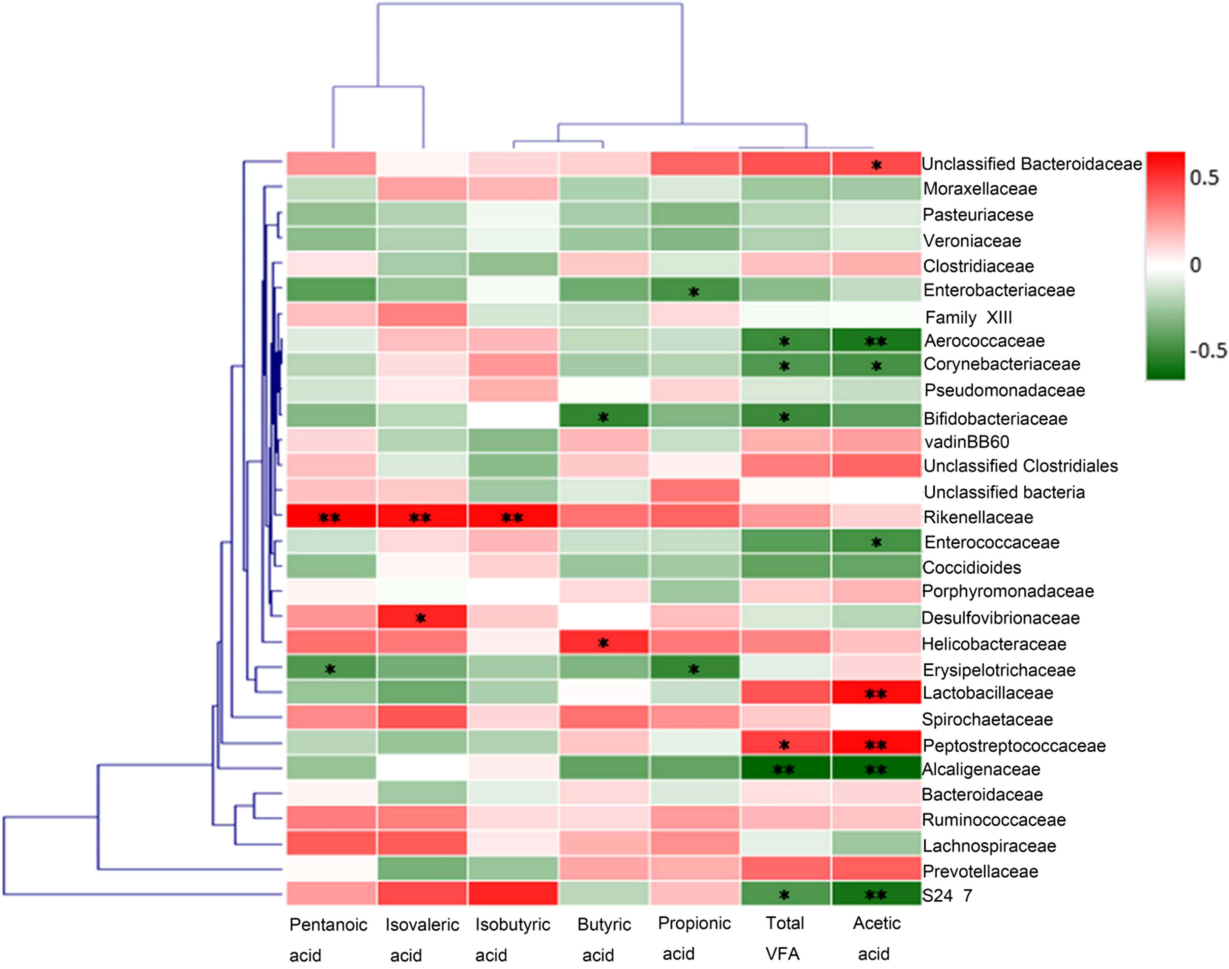
Correlation analysis between the abundances of fecal Short-chain fatty acid and representative bacteria. At the level of operational taxonomic units (OTUs), results from Spearman’s correlation analysis showed that the abundance of bacteria including *Lactobacillaceae* and *Peptostreptococcaceae* were positively correlated with the level of acetic acid in rat fecal samples, while the abundances of bacteria including *Aerococcaceae* and *Alcaligenaceae* were negatively correlated with that of acetic acid. *p<0.05, **p<0.01.

In order to explore the potential sources of SCFAs, we evaluated the correlation between the levels of acetic acid in rat fecal samples and the abundance of some representative bacteria. At the level of operational taxonomic units (OTUs), results from Spearman’s correlation analysis showed that the abundance of bacteria including *Lactobacillaceae* (*r*_s_ = 0.488) and *Peptostreptococcaceae* (*r*_s_ = 0.494) were positively correlated with the level of acetic acid in rat fecal samples, while the abundance of bacteria including *Aerococcaceae* (*r*_s_ = -0.467) and *Alcaligenaceae* (*r*_s_ = -0.528) were negatively correlated with that of acetic acid (**Figure 7**). Therefore, *Lactobacillaceae* and *Peptostreptococcaceae* appeared to contribute significantly to the production of acetic acid in rat intestine.

### Preterm rats in the normal control group and cognitive impairment group had significantly differed levels of blood soluble factors

**Figure 8:**
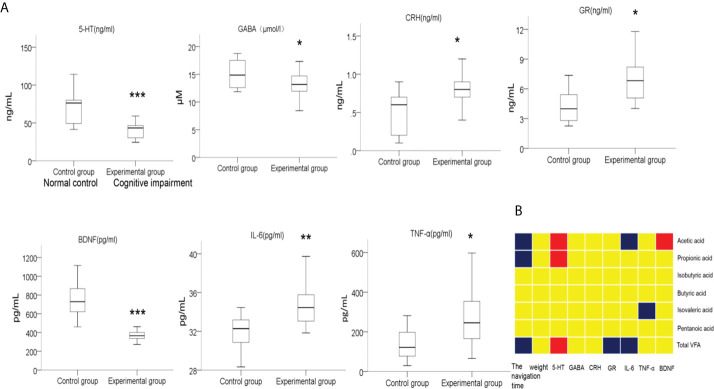
Comparison on the levels of serum factors between two groups of rats, and the correlations between the levels of these serum factors and the abundances of fecal SCFAs. **(A)** Blood samples were collected after the MWM tests from the rats in the normal control group and the cognitive impairment group. The levels of 5-HT, GABA, BDNF, GR, CRH, IL-6, and TNF-α in serum samples were determined by ELISA. n = 10 for the normal control group; n = 21 for the cognitive impairment group; *P < 0.05, **P < 0.01, ***P < 0.001, between the two groups. **(B)** The correlations between the abundances of total fecal SCFAs or the indicated specific SCFAs and the levels of serum factors, navigation duration, and rat body weight were analyzed, and the results are shown in the block chart. Red, positive correlation; blue, negative correlation; yellow, no correlation.

The normal function of the microbiota-gut-brain axis, the major bridge between the brain and intestinal microflora, is regulated by soluble factors like neurotransmitters, neuroendocrine factors, and pro-inflammatory cytokines (Liang et al., 2018; Cryan et al., 2019; Sun et al., 2020). We then compared the serum levels of soluble factors including 5-HT, GABA, BDNF, GR, CRH, IL-6 and TNF-α in preterm rats from the normal control group and cognitive impairment group. As shown in **Figure 8A**, the levels of 5-HT, GABA, and BDNF in the blood samples from the premature rats with cognitive impairment were significantly lower than that from the normal control group, while the serum levels of GR, CRH, IL-6, and TNF-α were significantly up-regulated in rats with cognitive problem. Thus, the alteration in blood levels of neurotransmitters, neuroendocrine factors, and pro-inflammatory cytokines was associated with the cognitive impairment in premature rats.

In addition, we also analyzed the correlation between the abundance of fecal SCFAs and the concentrations of these soluble factors, as well as the navigation duration and rat body weight. As shown in **Figure 8B**, the abundance of total SCFAs, acetic acid, and propanoic acid in fecal samples positively correlated with the concentration of serum 5-HT, while negatively correlated with the navigation duration in MWM tests, suggesting the alleviating effects of SCFAs for cognitive impairment. Moreover, the abundance of total SCFAs negatively correlated with the serum contents of GR and IL-6. Similarly, the levels of acetic acid and isovaleric acid were negatively correlated with the levels of the pro-inflammatory factors IL-6 and TNF-α, respectively, further supporting the anti-inflammation effects of certain SCFAs.

## Discussion

Accumulating evidence is supporting the notion that gut microbiota modifications play a crucial role in the dysfunction of cognitive ability in human through regulating the microbiota-gut-brain axis (Liang et al., 2018; Cryan et al., 2019; Zhu et al., 2020). How the intestinal microflora in preterm infants with cognitive impairment differs from the normal infants and how this axis is reregulated are not well understood so far. Here, we established a model of preterm rats with cognitive impairment by LPS administration in pregnant mother rats and confirmed the cognitive brain injury by performing the MWM navigation tests and HE staining of the hippocampus neurons. Compared with the normal control rats, the rats with cognitive impairment displayed significantly differed constitutions of intestinal microflora, as evidenced by significantly reduced richness and diversity of intestinal microflora, as well as significantly increased abundance of *Proteobacteria* in the cognitive impairment group. In addition, the abundance of total SCFAs and acetic acid was significantly lower in feces of rats with cognitive impairment. Furthermore, we identified that the changes in serum levels of a serial of soluble factors were associated with cognitive impairment in rats.

The balance of intestinal microbial richness and diversity is essential to maintain the health of the host (Berrington et al., 2014; Wang and Kasper, 2014; Gareau, 2016). Keller et al. established a significant correlation between the change in the intestinal flora and the abnormal development of the cognitive function (Bruce-Keller et al., 2017), which could be traced back to early postnatal brain development. The critical period of brain development is from 20 weeks of pregnancy to 3 years after birth, and the colonization of intestinal flora also occurs in the critical window of brain development. Also, the early postnatal brain development track overlaps with the acquisition and recombination of intestinal flora, suggesting a “time window” effect on intestinal flora and brain development (Berrington et al., 2014; Wang and Kasper, 2014; Gareau, 2016). Mayer et al. scanned the brain structure by magnetic resonance imaging (MRI) and found that the intestinal flora varied with different connections between brain regions (Mayer, 2011). This finding suggested that gut bacteria may help shape the brain structure during brain development. In this study, we found that the abundance and diversity of intestinal microflora in preterm rats with cognitive impairment were significantly reduced, which was consistent with the findings of the previous studies (Crumeyrolle-Arias et al., 2014; Desbonnet et al., 2015).

At the phylum level, we found that the *Proteobacteria* of the premature cognitive impairment rats was significantly higher in abundance than that of the normal control group. *Proteobacteria* accounts for only a small part of the intestines of healthy people (Faith et al., 2013). Once its abundance increases excessively, it will reduce the colonization ability of internal inherent flora against foreign pathogens, and further promote the inflammatory response and pathogen invasion. Therefore, the enrichment of *Proteobacteria* is also considered as a sign of intestinal flora disorder, which has potential diagnostic value (Shin et al., 2015). A study from Italy found that the relative abundance of intestinal *Proteobacteria* increased in patients with mild cognitive impairment (Cattaneo et al., 2017). Our further analyses on the intestinal flora at the level of order, family and genus indicated that the abundance of probiotics such as *Prevotella, Alitipes, Bacteroides*, and *Lactobacillus* decreased significantly, while the relative abundance of harmful bacteria such as *Staphylococcaceae, Enterococci, Oligella and Psychrobacter* increased significantly. Similarly, Berrington et al. found that the level of specific anaerobes in preterm infants decreased, while the level of facultative anaerobes in *Enterobacteriaceae* and *enterococcaceae* increased (Berrington et al., 2014). Moreover, Scheperjans et al. found that the abundance of *Prevotella* and *Lactobacillus* in patients with nervous system diseases was significantly lower than that in normal people (Scheperjans et al., 2015). Furthermore, the abundance of *Proteus* and *Bacteroides* in children with cognitive impairment was reported to be significantly lower than that in normal children (Srikantha and Mohajeri, 2019). Taken together, our and others’ results suggested that the changes in structure of intestinal microflora in preterm infants contributed substantially to their cognitive impairment.

At present, the mechanisms of intestinal microflora changes affecting cognitive development are not well elucidated. It was showed that the SCFAs produced by intestinal microflora has a significant effect on host physiology, including brain function and behavior (Selkrig et al., 2014). Supplement of bacteria that can produce SCFAs to germ-free mice enabled the normal functions of the blood brain barrier (Braniste et al., 2014), suggesting the beneficial role of SCFAs in maintaining the function of the blood brain barrier. In this study, the levels of total SCFAs and acetic acid in the cognitive impairment group were significantly lower than that in the normal control group. However, more studies with clinical samples are required to further substantiate the diagnostic value of these biomarkers. In addition, we analyzed the correlations between the abundances of SCFAs and intestinal representative flora in rat fecal samples. We found that *Lactobacillaceae*, *Peptostreptococcaceae* and acetic acid were positively correlated, while *Enterococcaceae*, *Aerococcaceae*, *Alcaligenaceae* and acetic acid were negatively correlated in fecal abundances. *Lactobacilli* are considered as traditional beneficial bacteria, and it can produce acetic acid with *Bacteroides* under the effects of certain specific bacteria (Slavica et al., 2015). It was showed that *Lactobacillus* intervention can improve the cognition of patients with Alzheimer’s disease, while the acetate intervention can improve the cognition of the disease mice (Agahi et al., 2018), suggesting the links between the decrease abundance of acetic acid and/or *Lactobacillus* and cognitive impairment. Additionally, it was found in animal experiments that the autism-like behavior of rats could be caused by a high concentration of propionic acid in the brain chamber (Ossenkopp et al., 2012). Liu et al. found that the concentration of butyrate in feces of autistic children is low (Liu et al., 2019). However, we found that there was no significant difference in the content of propionic acid and butyrate between the cognitive impairment group and the normal control group, which maybe affected by a variety of complex factors, such as individual differences in experimental animals, sample size and experimental environment. Collectively, we demonstrated that differed structure of intestinal microflora, together with significantly reduced abundances of SCFAs, contributed markedly to the cognitive impairment in preterm newborn rats.

As a major bridge between the brain and intestinal flora, the gut-brain axis is essential for the stability of intestinal flora and the normal function of the brain (Cryan et al., 2019; Sun et al., 2020; Zhu et al., 2020). In this study, the levels of 5-HT, GABA, and BDNF in the blood of premature rats with cognitive impairment decreased, while that of GR, CRH, IL-6, and TNF-α increased, in comparison to the control group. 5-HT and GABA are critical neurotransmitters that can protect cognitive function. Most of the 5-HT was synthesized in the intestine, and the content of 5-HT in the blood of the intestinal “germ-free” mice was significantly lower than that of the normal mice (Fung et al., 2019). Consistent with our results on the correlation of a lack in 5-HT and cognitive impairment, it has been shown that an increase in the brain 5-HT concentration with the use of *Lactobacillus helveticus* NS8 can improve the cognitive function of rats (Liang et al., 2015). Bravo et al. demonstrated that *Lactobacillus* regulates the emotional behavior and the expression of the central GABA receptor in mice through the vagus nerve (Bravo et al., 2011). In this study, we found that the content of GABA in the blood and the abundance of *Lactobacillus* in fecal samples decreased, which may be potential factors affecting the cognitive function of premature infants.

Several studies have shown that antidepressants increase the level of BDNF in plasma and improve the symptoms of depression (Molendijk et al., 2011; Zhou et al., 2017). In addition, Sudo et al. found that the expression of BDNF in the cerebral cortex and hippocampus of germ-free mice decreased significantly (Sudo et al., 2004), which might affect emotion, behavior, and sensation. In the present study, we found that the level of BDNF in rats with cognitive impairment was significantly lower than that in the normal control group, suggesting that the decrease in BDNF content also underlies the cognitive impairment in premature rats. Intestinal microflora regulates the HPA axis of the host, which is simultaneously regulated by CRH and GR at the same time (Sudo, 2006). The elevated content of *Proteus* causes intestinal inflammation and chronic inflammatory immune stress and increases the activity of the HPA axis (Sudo, 2006). HPA axis is crucial for learning and memory, and the disorder can lead to damage to hippocampus memory (Sudo, 2006). Golubeva et al. found that prenatal stress causes strong HPA axis response and cognitive dysfunction in adult male offspring rats (Golubeva et al., 2015). In line with these findings, we showed here that the abundance of fecal *Proteus* increased along with the decreased diversity and richness of intestinal microflora, while the levels of GR and CRH in serum were significantly higher than those in the normal control group. Therefore, we speculated that the increase in *Proteus* abundance might lead to the disorder of intestinal flora, inflammation, and chronic inflammatory stress reactions, which is a critical cause of cognitive impairment in preterm delivery.

The inflammatory cytokines can pass through the blood circulation and the blood-brain barrier to the brain endothelial cells, where they induce immune responses in brain and cause cognitive impairment. Based on the study conducted on aseptic mice, intestinal microflora exerts a significant impact on the function of immune system, and the diversity of intestinal microflora is positively correlated with the stability of the immune activity (Kuhn and Stappenbeck, 2013). Strikingly, upregulated levels of IL-6 and TNF-α in serum of patients with depression or Alzheimer’s disease were identified (Dowlati et al., 2010; Gaur and Agnihotri, 2015; Ng et al., 2018), suggesting a role of pro-inflammatory cytokines in promoting cognitive disorders. Consistently, we also showed the potential link on the occurrence of cognitive impairment and the elevation of IL-6 and TNF-α in peripheral blood of preterm rats, which might be induced by the disrupted intestinal microflora. However, the correlation between these changes in serum cytokine levels and the imbalance in the distributions of intestinal microflora still needs to be further investigated.

Based on the relatively limited sample size of each group in this study, in summary, the intestinal microflora structure of preterm rats with cognitive impairment changed significantly. The richness and diversity of rats in the cognitive impairment group were significantly decreased in comparison to that of the control group. Additionally, the abundances of probiotics such as *Prevotella*, *Lactobacillus*, *Bacteroides*, and *Alistipes* significantly reduced, and that of harmful bacteria including *Staphylococcaceae, Enterococci, Oligella and Psychrobacter* markedly increased. Moreover, the levels of total SCFAs and acetic acid in fecal samples of rats with cognitive impairment decreased. Furthermore, we found that the cognitive disorder and altered intestinal microflora structure in preterm rats were associated with decreased levels of 5-HT, GABA, and BDNF, and increased levels of GR, CRH, IL-6, and TNF-α in serum. Although more underlying molecular mechanisms need to be further investigated in a larger sample size, our current work supports the gut-brain axis, and provides valuable insights on targeting the modulation of intestinal microflora and the metabolites in treating the newborn with cognitive impairment.

## Data availability statement

The raw sequence reads from the metagenomic library was deposited in the Short Read Archive (SRA) of the GenBank database with accession no. PRJNA850563. The data set supporting the results of this paper are included in the main body of the article and in the **Supplementary Material**.

## Ethics statement

The animal study was reviewed and approved by Jiangsu University Ethics Committee.

## Author contributions

All authors participated in the design, interpretation of the studies and analysis of the data and review of the manuscript; WZ and HL contributed to the conception and design; XL, ZX, YQian, SW, and YQiao contributed to the provision of study materials or patients; XL, ZX, YQian, and SW contributed to the collection and assembly of data; XL, ZX, and YQiao contributed to the data analysis and interpretation. All authors contributed to the article and approved the submitted version.

## Funding

Zhenjiang Social Development Project [SH2019055].

## Conflict of interest

The authors declare that the research was conducted in the absence of any commercial or financial relationships that could be construed as a potential conflict of interest.

## Publisher’s note

All claims expressed in this article are solely those of the authors and do not necessarily represent those of their affiliated organizations, or those of the publisher, the editors and the reviewers. Any product that may be evaluated in this article, or claim that may be made by its manufacturer, is not guaranteed or endorsed by the publisher.

## Supplementary material

Supplementary Table 1 Comparison on MWM navigation test results of rats in the two groups.

Supplementary Table 2 Comparison of the intestinal flora richness and diversity indexes ( $\bar{x}$ ± s) between the two groups.

Supplementary Table 3 Comparison on the distributions ( $\bar{x}$ ± s, %) of the predominant intestinal microflora at the phylum level in the two groups.

Supplementary Table 4 Comparison on the distributions [M(P_25_, P_75_), %] of the dominant intestinal microflora at the order, family, and genus levels in the two groups.

Supplementary Table 5 Comparison on the distributions [M (P_25_, P_75_), %] of fecal SCFAs between the two groups.
