# Supplementary figures and images for: Changes in intestinal microflora and its metabolites underlie the cognitive impairment in preterm rats
Source: Front Cell Infect Microbiol. 2022 Aug 19;12:945851. doi: 10.3389/fcimb.2022.945851 (PMC9437323; doi:10.3389/fcimb.2022.945851)

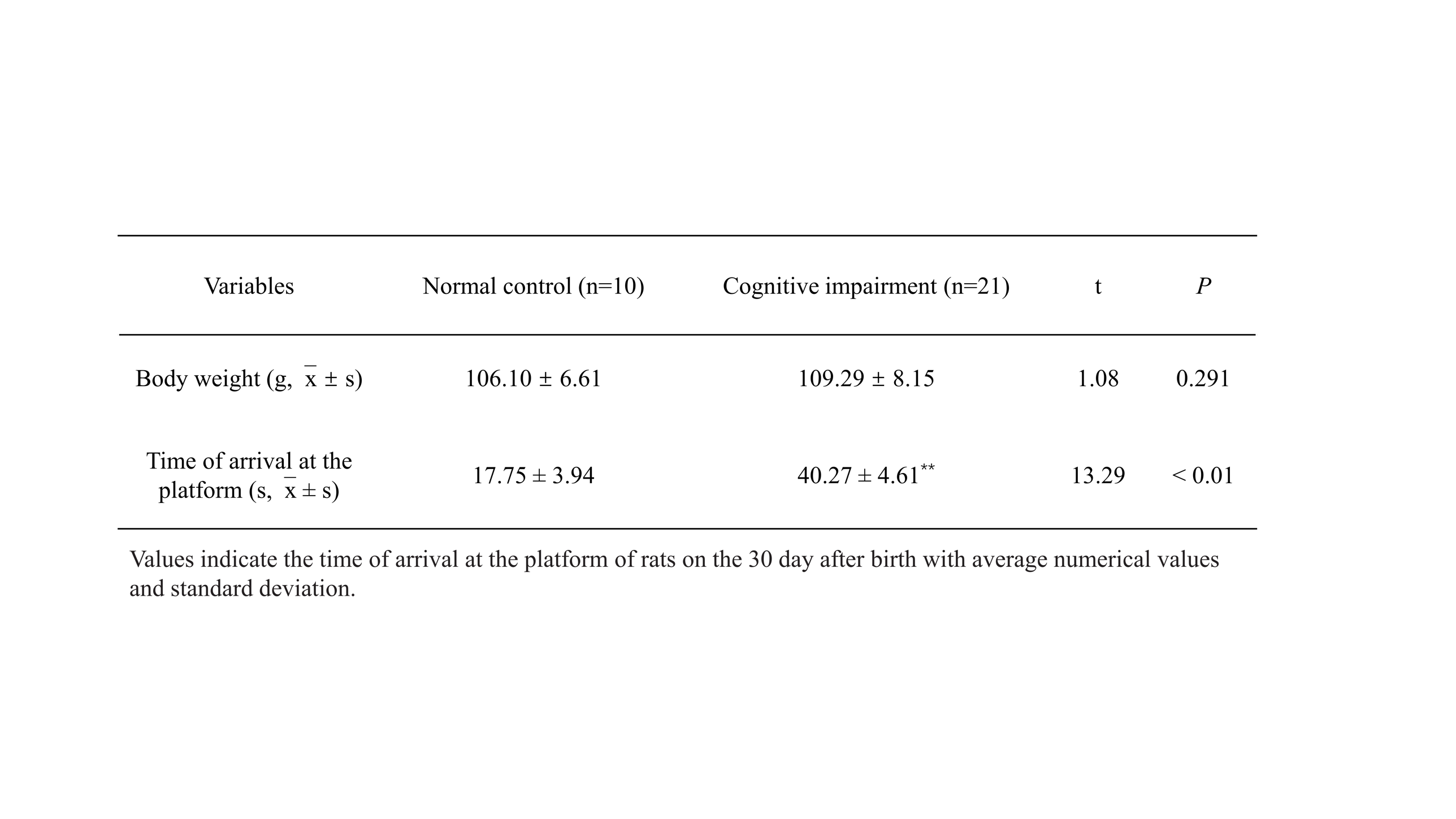

Supplement: Supplementary Table 1 — Comparison on MWM navigation test results of rats in the two groups. [file Image_1.jpeg]

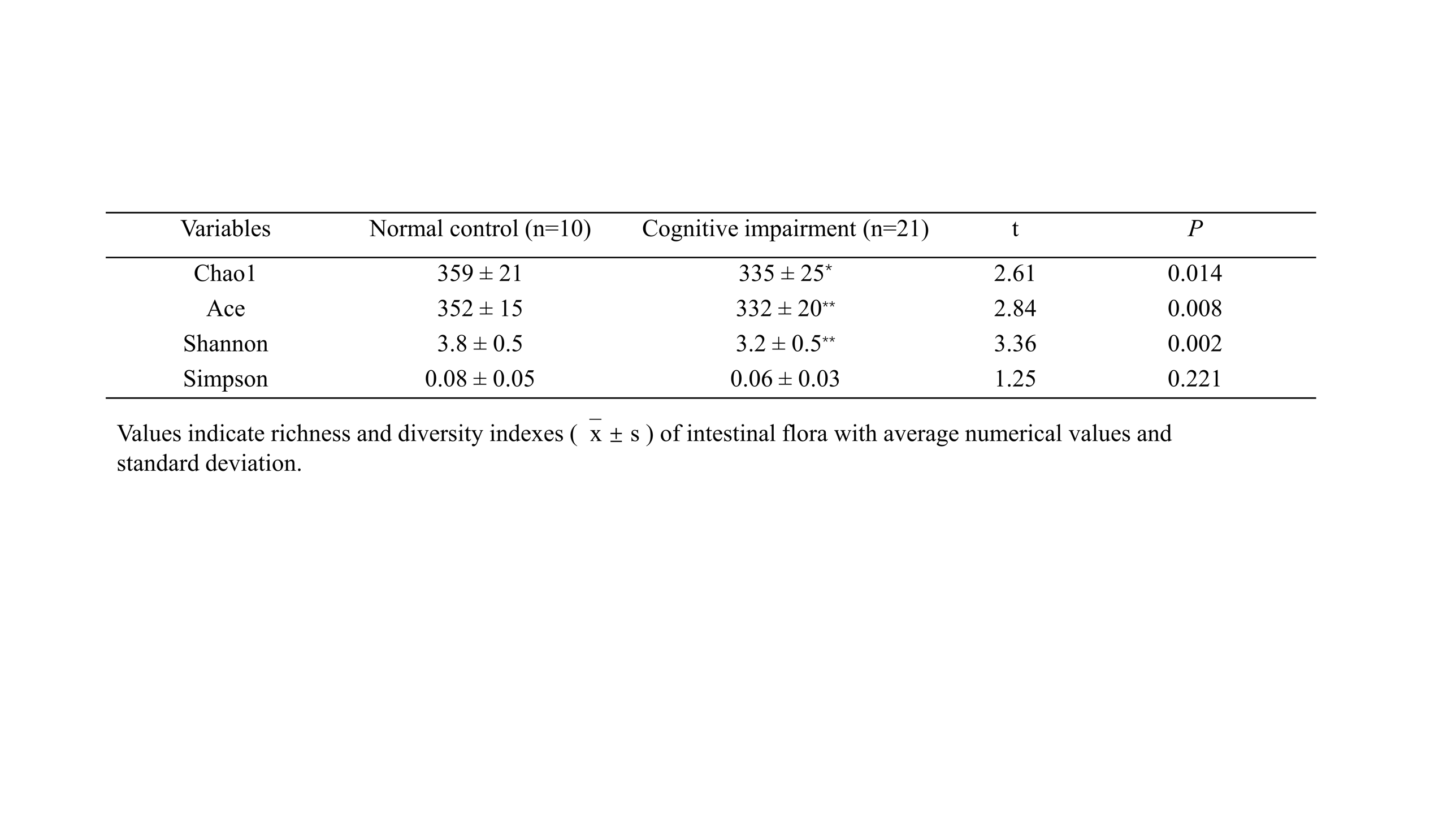

Supplement: Supplementary Table 2 — Comparison of the intestinal flora richness and diversity indexes ( x¯ ± s) between the two groups. [file Image_2.jpeg]

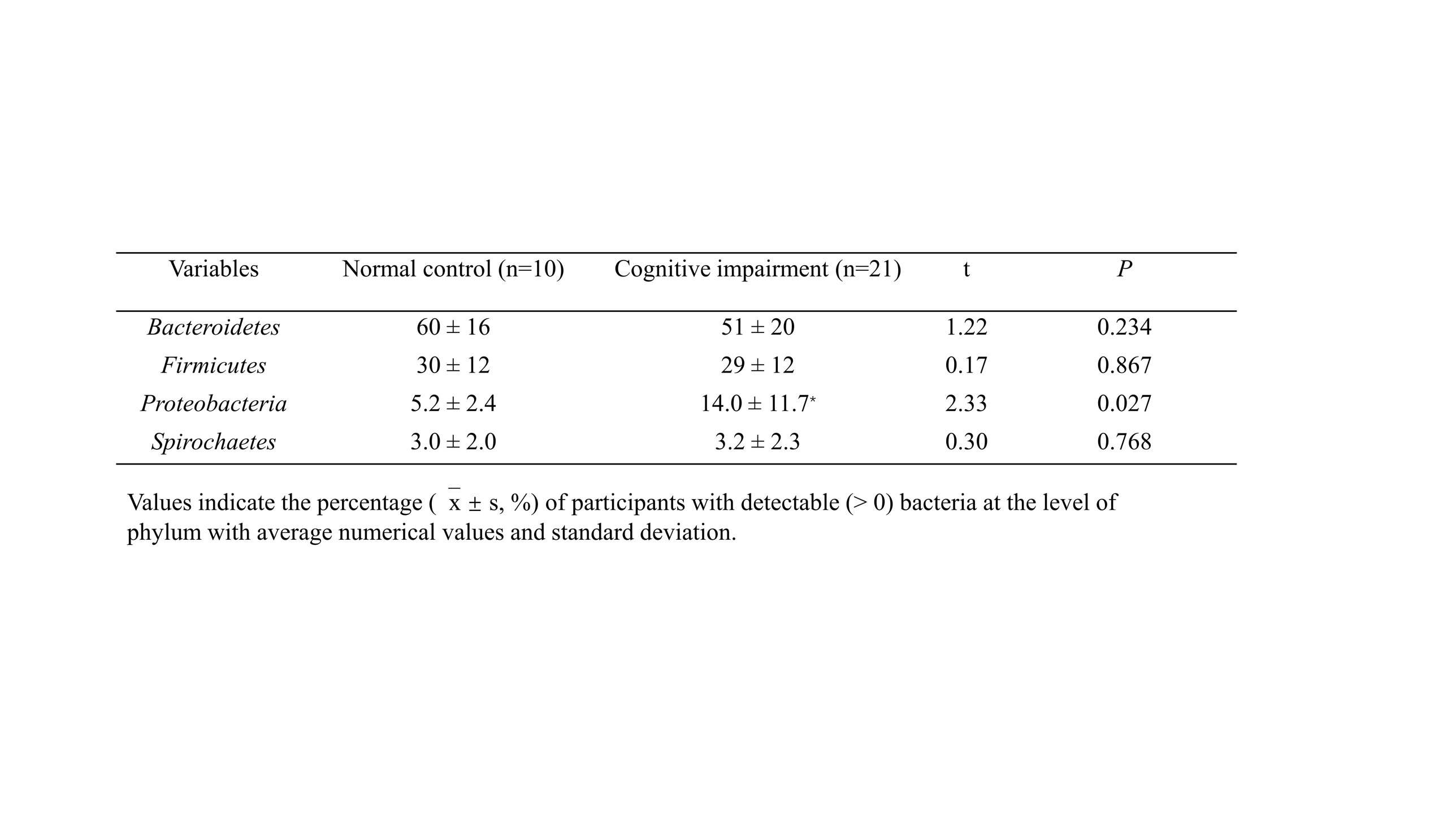

Supplement: Supplementary Table 3 — Comparison on the distributions ( x¯ ± s, %) of the predominant intestinal microflora at the phylum level in the two groups. [file Image_3.jpeg]

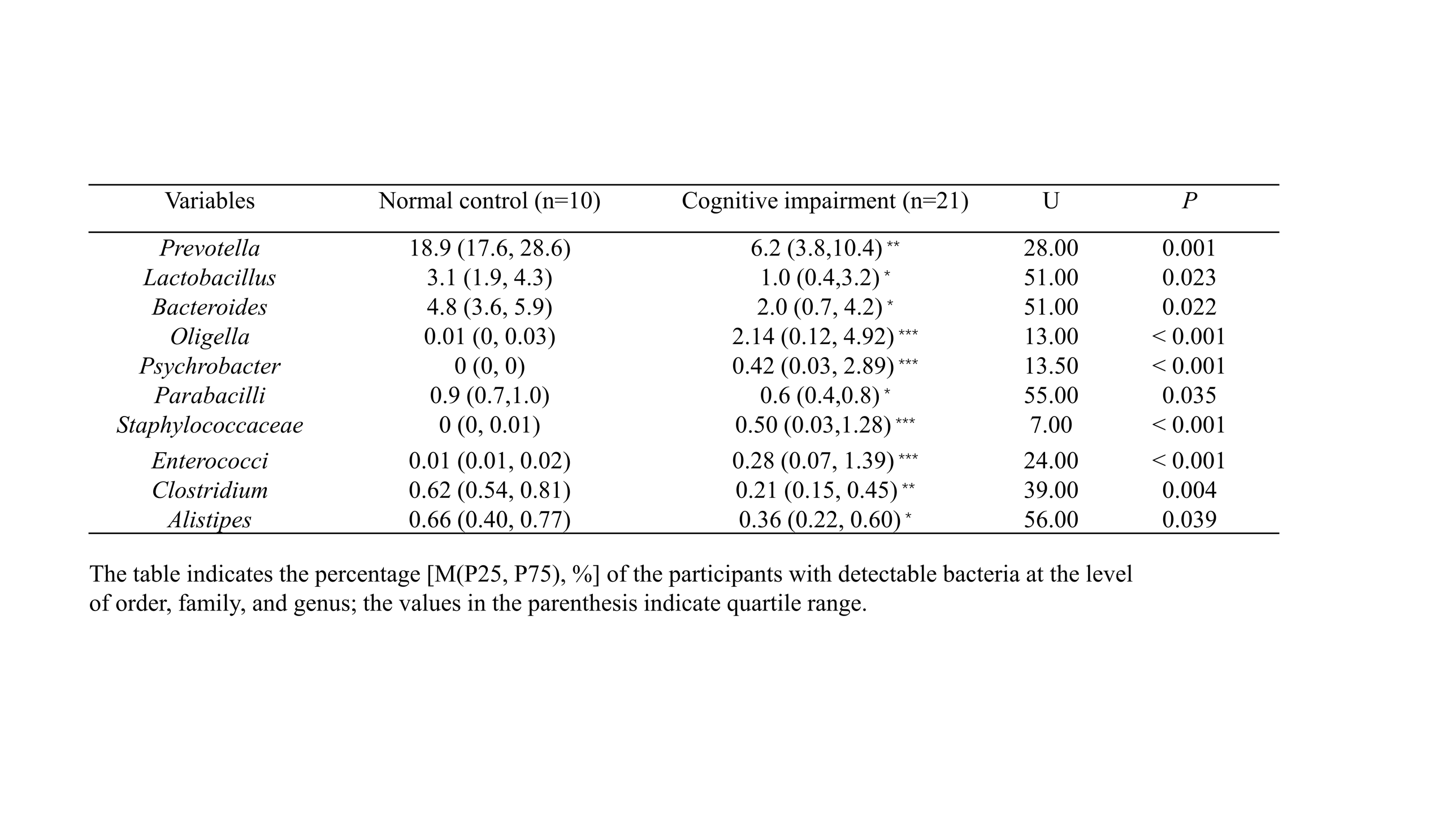

Supplement: Supplementary Table 4 — Comparison on the distributions [M(P25, P75), %] of the dominant intestinal microflora at the order, family, and genus levels in the two groups. [file Image_4.jpeg]

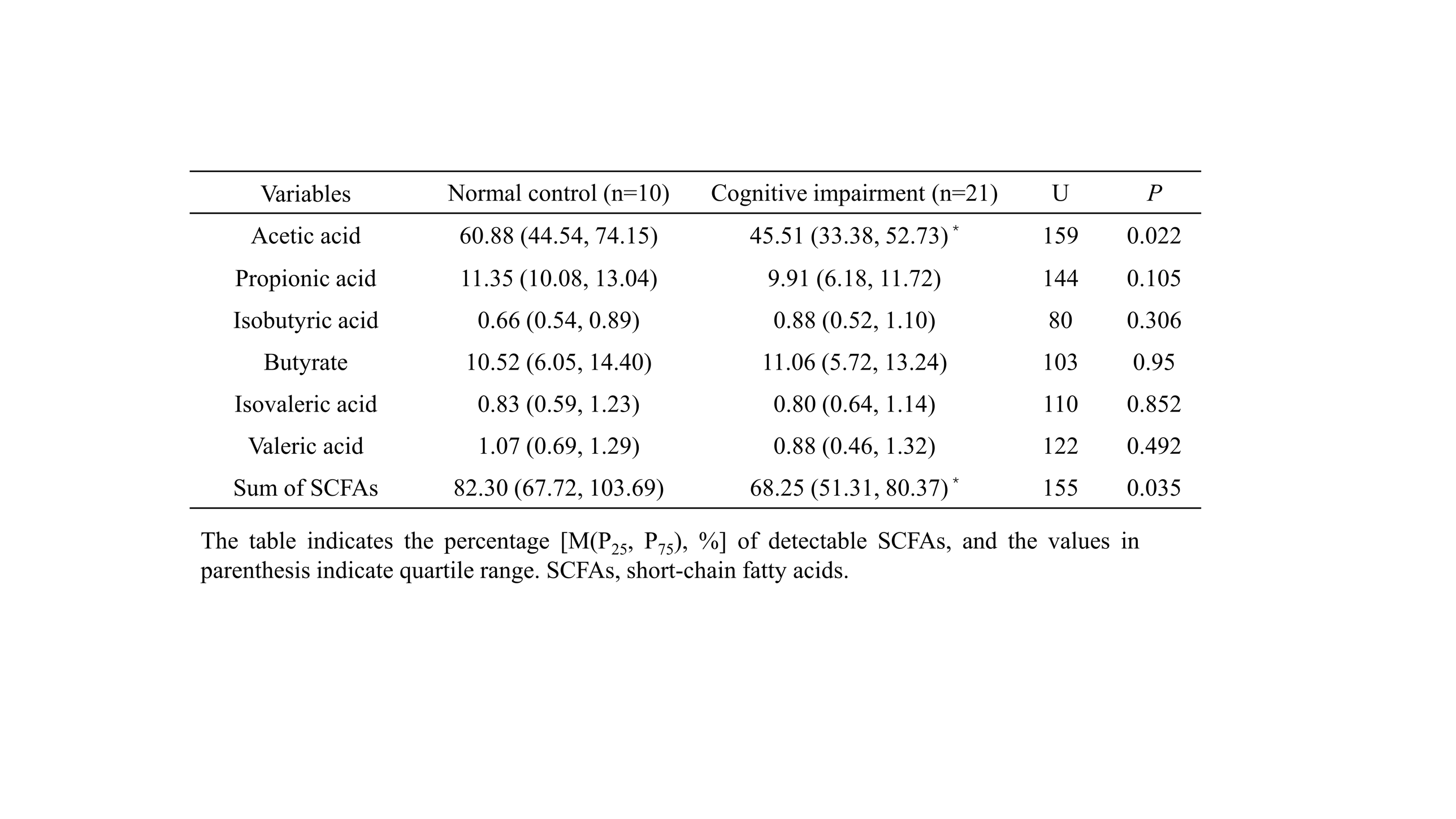

Supplement: Supplementary Table 5 — Comparison on the distributions [M (P25, P75), %] of fecal SCFAs between the two groups. [file Image_5.jpeg]
